# Building Fine Houses on Sand

**Published:** 1883-08

**Authors:** C. M. Wright

**Affiliations:** Cincinnati


### Building Fine Houses on the Sand.

PROF. BY C. M. WRIGHT, CINCINNATI.

The human teeth, from the firmness of their bony sockets and foundations, the strength of their arches, the hardness of the covering of their exposed parts, and the structure of this enamel, as shown by the microscope, were no doubt originally intended to do a great amount of hard work in the preparation of all sorts of hard substances for reception by the stomach. They were designed as millstones to grind up the food that is afterwards dissolved and absorbed in its journey through the alimentary track. Civilization, with its flour mill, its patent range, its cuisine, has taken away the necessity for hard work on the part of the teeth, and they have naturally fallen into disuse, and are liable to decay and disease. Aches and pains, discomfort, and disfiguration of the human face have followed—are, indeed, the natural sequence of the disuse or perversion of use of organs. Perfect sets of natural teeth, complete in number, perfect in form and position, untouched by decay, are *very rarely seen* at the present time *in any civilized country.* Dentists, whose occupation it is to battle with the tendency of the times, and to combat the diseases of these organs—to save them from the consequences of civilization, are very numerous, are very active as a profession, are very ingenious, and are in short, a *necessity* of the age. They supply an urgent demand of the civilized world. The more cultivated the community, the more numerous and prosperous are the dentists. Every observant traveler knows this. The cultivated community therefore is interested in the questions—Can or does the dentist successfully combat this great tide of steadily progressing evolution in the teeth, that dates back to the cradle times of civilization? Does he successfully hinder the ravages of decay, the tendency toward which *is born with every child*—the *predisposition* toward which *every tooth germ* contains? Can the dentist of to-day successfully cope with a disease, the seeds of which were planted generations ago? Does he, as he passes his lenses over the enamel of the teeth read in this dark spot, the record of an eruptive disease of the great grandfather when he was a boy, or in that white speck, the record of a grandmothther's broken health? I do not think that the profession, as a rule, bear these things in mind, for if it did, the practice would certainly be different, especially in the United States of America, where we are so boastful about the superiority of "American dentistry." Some years ago, I wrote an article for one of the dental journals, on "Gold as a Temporary Stopping." The statements were based on careful observations, extending over a number of years, and under favorable circumstances. My article was vigorously attacked by one of the most earnest and enthusiastic laborers in the dental field. I appreciated to the full his enthusiasm and his *theory*, in the series of replies to articles on the same subject—and since my return from a ten years' practice in Europe, and after a more intimate acquaintance with the *theories* of both dentists and patients here in Cincinnati, I can understand how popular his views are at home. Dentists teach that gold is the most permanent filling material, and that when they wish to save a tooth "permanently" they employ gold. Patients believe that *gold* and *permanency* are synonomous terms. Surprise and indignation is expressed by intelligent people when gold does not save a tooth *permanently*. "Why," says one, "they say Dr. A is a fine dentist, but I know that he is not, for here is a $10 *gold* filling that lasted only two years;" or, "please fill my teeth with *gold*, for I wish to keep them, and don't always want to be running to the dentist;" or, "I'd rather submit to the suffering now, and have them filled *permanently* with *gold*, than to lose my teeth." Dentists talk in open meeting in the same strain: "I filled the tooth temporarily with oxy-phosphate, intending later to insert a *permanent* filling" (meaning gold); or, in their offices—"Why, madam, these fillings made by Dr. B, are only temporory, and should be replaced with permanent gold fillings." Gold, then, is a permanent filling! Gold, then, which is simply an inert metal, with no therapeutic effects, unless the irritation produced by the transmission of hot and cold sensations to the nerve of a tooth may happen to be just sufficient to cause the pulp to throw out new protective material—gold, in the form of foil or crystals, pushed and wedged and pounded into a sensitive cavity in a living tooth, will *permanently* arrest disease in a tooth *predisposed* from inheritance and *constantly exposed* to decay! During every second of existence, from early morn till night, during the hours when other organs rest, the teeth are exposed to disintigrating ferments. While beauty sleeps her teeth are too frequently decaying. This is not pleasant, but true. No one who has studied the wonderful processes of the complex disease--decay of the teeth; who considers the *pedigree* of the tooth, its own *life history*, the subtle *acids* and *ferments*, the *micro-organisms*, can help the feeling that, the most ridiculous idea in the dental world is the connection of *permanency* with gold, in a human tooth, in a living person's mouth. Gold is a beautiful metal. It is a useful metal. It is difficult to obtain, difficult to fashion, and consequently an expensive metal. In dentistry, it is, when pure enough, a valuable and strong material upon which to mount artificial teeth, and soft and pliable enough when specially prepared for the purpose, to allow the skillful dentist to force it very tightly into cavities and crevices in strong human teeth, and cohesive enough to allow him to build it out into the shape of broken corners of teeth, but it has no preservative virtue aside from the mechanical closure of a cavity, and, as it is difficult to manipulate, a large majority of cavities in locations difficult of access are not thoroughly stopped. It does not hermetically seal the cavities, and decay is sometimes fostered, rather than arrested by fine looking gold fillings. Conscientious and *thinking* dentists all *know* this to be true, and yet habit, the desire to satisfy the expectations of eager patients, and the lack of *independent* honesty, cause many to allow this fatal error to grow, and they even continue to sow the seed, by their practice and teaching.

GOLD IS A BEAUTIFUL TEMPORARY STOPPING for decayed teeth. *No material now known is permanent* in the sense of arresting permanently decay of the teeth. The sooner the dental profession begin to impress these truths on the minds of the people, and the sooner the people fully appreciate the truth, and the great facts that lie under it, the sooner will the forceps be *permanently* laid aside, and *artificial teeth* become less frightfully common. What a crippled set of people we should consider ourselves if false noses, or false legs or arms were but one half as common as false teeth!

Scientific dentistry is not simply "skill in filling teeth with gold." Scientific dentistry employs means to an end, and is not ruled by fashions or false proverbs—and to scientific dentistry must the people look for help. Gold can be built upon a rock and remain —but only the foolish would build upon the sand, where every wave threatens to wash away the structure.

Wise patients would visit an honest dentist several times a year for the purpose of having their teeth *cleaned* and examined, and attended to in time, and would feel ashamed to care less for their teeth, and the cleanliness of their mouths than they do for their finger nails or hair. Gentlemen boast that they take care of their teeth, when they visit their dentist once in from two to five years; but they visit their barbers regularly, and spend ten fold as much time and money on their chins and heads without grumbling, as they give to scientifically educated dentists for the care of their teeth. This is not an evidence of the nicest refinement of civilization. Not that they should love the barber less, but that they should love the dentist more. This startling assertion can be proved—that in the highest circles of society in Europe and America, among ladies of elegant manners and refined feelings, and gentlemen of cultivated intellects and careful habits, and children of tender nurture and intelligent training, *ninety* in every hundred, or 900 in every 1000, have, not only teeth liable to *active* decay, and in different stages of disease, but *diseased gums, unhealthy oral secretions* or *unclean deposits of mixtures* of the *precipitations* from the saliva and food about the teeth; minute cleanliness being an extremely rare exception. Dentists are in a measure to be blamed for much of this carelessness, for they have not taught that monthly or bi-monthly visits should be made to remove tartar, and inspect the teeth, but have made heroic "operations" in gold, taken large fees, and *partially* guaranteed by word or inference that "now, after these large *permanent* operations in gold, your mouth will not require a dentists interference for months or years." Patients are taught this, I say, by good dentists. It is a delusion and a snare. It is all wrong. If large expensive fillings have been made, extra vigilance is required on the part of the patient, to save not only his teeth, but the dentist's work. If you should build a costly palace on the sands of the seashore, would you after paying the architect and builder leave it to the winds and waves? Would you not keep a constant watch to protect it from constant destructive tendencies? Would you not frequently want the builder's inspection and assurance that all was right? If a tooth, (that when healthy is so hard and firm), can be melted like snow before a summer's sun in a short time in your mouth, has received a $20 gold filling, you feel quite contented that the tooth has been saved. Does the erection of an expensive house on the sand, cause the foundation to turn to rock? Wise men build at little expense, and with little annoyance, light sea-side cottages or *tents* on the sands, and if they are undermined by an extra wave, or blown over by a high wind, they can be laughingly set up again at little trouble and expense.

The dentist of the future will stop up the holes made by disease in the teeth with "neatness and dispatch," using of the materials according to the condition of the teeth, aiming to avoid heroic or capital operations and large bills for single temporary operations in gold, and by frequent visitations, by careful and skillful regard for the cleanliness and purity of the surroundings, nurse the *teeth* that were stamped by disease so long ago, into comfortable and beautiful usefulness. This can be accomplished by dentistry. But, "gold is a temporary stopping," and *safety* and *cleanliness* demand *very frequent* attention from the *dentist.*

I have risked the statement in private conversation with dentists that, "I solemnly believe that if we, the dental profession, should be suddenly forbidden the use of gold for filling teeth, dentistry would soon lose its *trade* character, and become gradually established on a *professional* basis, for we should then be put to our wit's ends to discover therapeutic measures, in the field of Materia Medica, to arrest decay, and should be doctors indeed." I would not risk this statement in print, knowing that when a dentist raises his voice even to a whisper against "gold for filling teeth," he is immediately set down as *incompetent* to wield the plugger and mallet—incapable of deftly applying rubber dam—*unable* to skillfully carve dentine and enamel, and to build out with gold, artistic (?) corners of tooth crowns, and to *finish* the same beautifully. The *reputation* of skill in these "operations" is very important to a dentist. It *is dentistry* in the popular estimation. I know of a bi-cuspid in a French gentleman's mouth that for six or seven years did excellent service as a masticator--surrounded by healthy gum tissue----presenting a good and clean and clear appearance, that had a rather large cavity in its posterior portion and an open pulp chamber and canals. An English dentist had cleaned these canals and cavities, had given the patient prescriptions for a disinfecting dressing, and a mastic preparation, and minute directions how to remove and apply these dressings and mastic on cotton. The French gentleman cleaned the tooth and filled the cavity with his cotton dressing and covered with mastic every morning and evening, and all was sweet and clean. Of course this required care and time, but the *lesson* is there. If an intelligent dentist should be cast away on an island where the inhabitants had decaying teeth, I can well imagine how he could, by the use of antiseptics and mastics (not plastics) alone, successfully combat decay in from 500 to 800 mouths, and this, by half hour sittings for each, once a month---provided his patients obeyed his instructions. This is fancy, but it is the *direction* that we, as a profession, should take to the goal, we talk about. When we are appreciated and paid for such visits, instead of for gold by the grain or square inch, we shall save more teeth than at present.

Do I condemn the employment of gold as a filling material? I answer with a shout, No! I condemn only the *promise* of *permanency* on the part of the dentist. This *promise* does harm. This *promise*, spoken or inferred, has been attached to gold, and has done infinite injury to the noble metal, to a noble profession, to a great work, to thousands of people. By the use of gold thousands upon thousands of teeth *have been saved*, but gold should never be thought of by patient or dentist, excepting as a *means* to an *end*.

"It will cost $10 with gold, and $3 with cheaper materials,"

and all that *this* implies is as unworthy of a true dentist, as, "it will be cheaper to bury your son than to cure him," would be from a physician, who was also undertaker—or who combined, as we do, trade interests with professional aspirations.

